# Vaccination Intention and Behavior of the General Public in China: Cross-sectional Survey and Moderated Mediation Model Analysis

**DOI:** 10.2196/34666

**Published:** 2022-06-20

**Authors:** Liuqing Yang, Lili Ji, Qiang Wang, Yan Xu, Guoping Yang, Tingting Cui, Naiyang Shi, Lin Zhu, Shixin Xiu, Hui Jin, Shiqi Zhen

**Affiliations:** 1 Department of Epidemiology and Health Statistic School of Public Health Southeast University Nanjing China; 2 Key Laboratory of Environmental Medicine Engineering, Ministry of Education School of Public Health Southeast University Nanjing China; 3 Jiangsu Provincial Center for Disease Control and Prevention Nanjing China; 4 Department of Immunization Planning Wuxi Center for Disease Control and Prevention Wuxi China

**Keywords:** vaccine, theory of planned behavior, attitude, subjective norms, perceived behavior control, moderator, mediation

## Abstract

**Background:**

Promoting vaccination and eliminating vaccine hesitancy are key measures for controlling vaccine-preventable diseases.

**Objective:**

We aimed to understand the beliefs surrounding and drivers of vaccination behavior, and their relationships with and influence on vaccination intention and practices.

**Methods:**

We conducted a web-based survey in 31 provinces in mainland China from May 24, 2021 to June 15, 2021, with questions pertaining to vaccination in 5 dimensions: attitude, subjective norms, perceived behavioral control, intention, and behavior. We performed hierarchical regression analysis and structural equation modeling based on the theory of planned behavior—in which, the variables attitude, subjective norms, and intention each affect the variable intention; the variable intention mediates the relationships of attitude and subjective norms with behavior, and the variable perceived behavioral control moderates the strength of this mediation—to test the validity of the theoretical framework.

**Results:**

A total of 9924 participants, aged 18 to 59 years, were included in this study. Vaccination intention mediated the relationships of attitude and subjective norms with vaccination behavior. The indirect effect of attitude on vaccination behavior was 0.164 and that of subjective norms was 0.255, and the difference was statistically significant (*P*<.001). The moderated mediation analysis further indicated that perceived behavioral control would affect the mediation when used as moderator, and the interaction terms for attitude (β=–0.052, *P*<.001) and subjective norms (β=–0.028, *P*=.006) with perceived behavioral control were significant.

**Conclusions:**

Subjective norms have stronger positive influences on vaccination practices than attitudes. Perceived behavioral control, as a moderator, has a substitution relationship with attitudes and subjective norms and weakens their positive effects on vaccination behavior.

## Introduction

Vaccines play a crucial role in protection against infectious diseases. Vaccination is an important component of both family and public health. However, confusion and misunderstanding still surround vaccines, even though they protect against a wide variety of organisms that cause disease, such as influenza, cervical cancer, hepatitis B, COVID-19, pneumonia, and rabies. A recent study [1] by the COVID-19 reaction team at Imperial College London, found that high vaccine hesitancy rates can significantly prolong the time required for nondrug interventions to maintain and decrease the mortality associated with COVID-19. Therefore, exploring the mechanisms behind vaccination behavior to reduce vaccine hesitancy and improve vaccination rates has become a key research topic. Research on vaccine acceptance suggests that individual decisions about vaccination behavior are much more complex and may involve emotional, cultural, social, spiritual, or political factors, as well as cognitive factors [2,3]. It has been demonstrated that theory-based behavioral interventions are more effective [4-9]. However, rather than building on the premise of theoretical models to test hypotheses, most studies [10-12] that have collected data to conduct exploratory studies of knowledge, attitudes, and beliefs or have focused on demographic factors related to vaccination practices.

Currently, the theory of planned behavior, which is one of the most commonly used psychological theories to explain health behavior, is considered to be the most suitable for explaining vaccination behavior [5,13-15], and has been used effectively as a theoretical framework for designing health behavior interventions [16,17]. Another framework—the health belief model—is also widely used in behavioral health fields; however, studies [18-20] have shown that the health belief model is more suited to description rather than explanation of health behavior and has weak predictive validity. Although Webb et al [21] found that theory of planned behavior–based interventions were more effective than those based on other theories, existing studies on theory of planned behavior have not been in-depth—a systematic review [22] demonstrated that most researchers did not address adaptive feedback, merely focused on intention as the outcome, and ignored mediating effects between intentions and behaviors. Yet, the purpose of the theory of planned behavior was to account for perceived behavioral control, which, as a representative of actual behavioral control, should have an impact on the overall model [23].

The beliefs and behavioral drivers of vaccination need to be studied to be able to develop better targeted intervention strategies. We aimed to confirm the theoretical validity and ability of the theory of planned behavior to explain vaccination practices.

## Methods

### Ethics Approval

The Wuxi Center for Disease Control and Prevention Ethics Committee approved this study (2020No10).

### Participants and Procedure

From May 24 to June 15, 2021, we conducted surveys in 31 provinces in mainland China using web-based questionnaires. We used convenience sampling. The link to the questionnaire was created through *Wen Juan Xing*, which is a platform dedicated to the creation and dissemination of questionnaires, and forwarded by the WeChat platform of the Jiangsu Provincial Center for Disease Control and Prevention. Written information was provided as a statement that could be read, which assured participants that the study was conducted on a voluntary basis and for research purposes only. All surveys were conducted in Chinese. (The surveys were translated into English by 2 researchers only for the purpose of this paper.) Questionnaires answered by people under 18 years and over 60 years were not analyzed. To prevent repeated submission of questionnaires, WeChat real name verification was required when using the link to fill out the questionnaire, and an IP address could only be used to submit a questionnaire once. Questionnaires completed in less than 60 seconds were automatically discarded. Questionnaires with selections at the same level of the Likert scale were also considered invalid.

The questionnaire (Multimedia Appendix 1) was used to collect demographic information (age, gender, ethnicity, usual place of residence, education level, annual household income, and whether respondents worked in the health care industry, residential status), chronic disease history, and information on self-assessment of health status.

### Theoretical Background

The theory of planned behavior is widely used to study intentions and behaviors. In this model, intentions are considered the most direct predictor of behaviors and are weighted based on attitudes, which is the degree to which behaviors are positively or negatively evaluated, and subjective norms, which is the pressure society places on implementing or not implementing behaviors. The effects of attitudes and subjective norms are mediated by perceived behavioral control, which is people's perceptions of their ability to perform a given behavior. When perceived behavioral control is accurate, it acts as a proxy for actual behavioral control, that is, the extent to which a person has the ability, resources, and other conditions required to perform the behavior.

In this study, the variable *attitude* represented people's positive or negative perceptions of the vaccine. The variable *subjective norms* referred to the expectations of family, friends, and physicians. The variable *perceived behavioral control* represented people's beliefs about barriers to vaccination (such as the time, cost, and side effects caused by vaccination). Each item was assessed using a 5-point Likert scale (Table 1).

**Table 1:** Translation of the questionnaire.

Dimension and questions		Scale
**Attitude**		1 (strongly disagree) to 5 (strongly agree)
	Q1. I think that vaccination is safe.	
	Q2. I think that vaccination is effective.	
	Q3. I think that vaccination is beneficial.	
	Q4. I think that vaccination is important.	
**Subjective norms**		1 (strongly disagree) to 5 (strongly agree)
	Q5. Did my family, doctors, and close friends think I should be vaccinated?	
	Q6. Will I do what they think I should do?	
	Q7. Can vaccination can protect close relatives from relevant vaccine-preventable diseases?	
**Perceived behavior control**		1 (strongly disagree) to 5 (strongly agree)
	Q8. The possibility of still being infected after vaccination would discourage me from getting vaccinated.	
	Q9. The exorbitant cost of vaccinating would stop me from getting vaccinated.	
	Q10. Vaccination causes a decline in autoimmunity.	
	Q11. Concerns about side effects of the vaccine stop me from getting vaccinated.	
	Q12. Difficulty in obtaining an appointment for vaccination would prevent me from getting vaccinated.	
**Intention**		1 (completely impossible) to 5 (completely possible)
	Q13. The possibility of considering getting vaccinated.	
	Q14. The possibility of trying to get vaccinated.	
	Q15. The possibility of actually getting vaccinated.	
**Behavior**		1 (completely impossible) to 5 (completely possible)
	Q16. How likely are you to go for a COVID-19 vaccine?	
	Q17. What is the possibility of getting an inﬂuenza shot this year?	
	Q18. What is the level of hesitation about vaccinating?	

### Hypotheses

#### Hypothesis 1

*Attitude* will have a positive association with vaccination *behavior*.

#### Hypothesis 2

*Subjective norms* will have a positive association with vaccination *behavior*.

#### Hypothesis 3

Vaccination *intention* will have a positive association with vaccination *behavior*.

#### Hypothesis 4

Vaccination *intention* will mediate the relationships of *attitude* (hypothesis 4a) and *subjective norms* (hypothesis 4b) with vaccination *behavior*.

#### Hypothesis 5

*Perceived behavior control* will moderate the strength of the mediated relationships of *attitude* (hypothesis 5a) and *subjective norms* (hypothesis 5b) with vaccination *behavior* via vaccination *intention*.

### Model Testing

Before structural equation modeling, confirmatory factor analysis was conducted to assess the reliability and validity of the constructs. Reliability was assessed by calculating the squared multiple correlation [24] and composite reliability [25]. We also examined parameter estimates and their associated *t* values, factor loadings, and the average variance extracted [26]. We established discriminant validity by calculating the square root of the average variance extracted for each latent variable. The error variances and modification indices of items were estimated.

### Model Fitting

The goodness-of-fit index (GFI), adjusted GFI, comparative fit index (CFI), and the root mean square error of approximation (RMSEA) were used to evaluate the model fit [27]. For GFI, CFI, and adjusted GFI, values closer to 1 are better, and values greater than 0.95 indicate relatively good fit; RMSEA values less than 0.06 indicate relatively good fit [27,28].

### Statistical Analysis

We used bootstrapping (5000 trials) to test mediator effects [29]. Hierarchical moderator regression was used to test moderation effects, and all variables were standardized to avoid multicollinearity [30]. The control variables were entered in the block 1 (gender, education level, health care occupation, annual household income, main living condition, self-evaluation of health, chronic diseases, past behavior), followed by the standardized value of the main effect (*attitude*, *subjective norms*, and *perceived behavioral control*) in block 2, and finally, the interactions and moderators (*subjective norms* * *perceived behavioral control*, *attitude* * *perceived behavioral control*) in block 3.

AMOS software (version 23; IBM Corp) was used to estimate the structural equation coefficients between latent variables in the model. Hierarchical moderator regression analysis was performed using SPSS software (version 23; IBM Corp). Statistical significance was set at *P*<.05.

## Results

### Participant Information

A total of 9924 participants (male: 5407/9924, 54.5%; female: 4517/9924, 45.5%) were included in this study (Table 2). Most respondents had a college degree or higher (7589/9924, 76.4%), and did not work in health care–related industries (7007/9924, 70.6%).

In this study, the proportion showing vaccine hesitancy (respondents who selected not sure, hesitant, and very hesitant for Q18) was estimated to be about 26.6% (2640/9924). Of the total sample, 77% (7643/9924) reported that they had received COVID-19 vaccinations, and 29.4% (2922/9924) had received the influenza vaccinations in the previous year. Of the women, 22% (992/4517) had received human papillomavirus (HPV) vaccinations. Of the total sample, of the majority believed that they would choose to receive COVID-19 (8614/9924, 86.8%) and influenza (3315/9924, 33.4%), vaccinations this year.

**Table 2:** Participant information.

Characteristic		Respondents (n=9924), n (%)
**Age group (years)**		
	18-24	2362 (23.8)
	25-34	3963 (39.9)
	35-44	2334 (23.5)
	45-54	1125 (11.3)
	55-59	140 (1.4)
**Gender**		
	Male	5407 (54.5)
	Female	4517 (45.5)
**Educational level**		
	High school graduate or below	2335 (23.5)
	College or equivalent	6822 (68.7)
	Master’s diploma or above	767 (7.7)
**Health care occupation**		
	Yes	2917 (29.4)
	No	7007 (70.6)
**Annual household income (US $)**		
	<16,000	4285 (43.2)
	16,000-32,000	4259 (42.9)
	32,000-80,000	1112 (11.2)
	>80,000	268 (2.7)
**Main living condition**		
	Living with others	9145 (92.2)
	Alone	779 (7.8)
**Self-evaluation of health**		
	Very bad	359 (3.6)
	Bad	242 (2.4)
	General	3168 (31.9)
	Well	3816 (38.5)
	Very well	2339 (23.6)
**Chronic diseases**		
	Yes	1275 (12.8)
	No	8649 (87.2)
**Influenza vaccination history (last year)**		
	Yes	2922 (29.4)
	No	7002 (70.6)
**COVID-19 vaccination history**		
	Yes	7643 (77.0)
	No	2281 (23.0)
**HPV^a^ vaccination history (n=4517)**		
	Yes	992 (22.0)
	No	3525 (78.0)

^a^HPV: human papillomavirus.

### Measurement Model and Fitting

The 18 items were found to be reliable and valid based on each item’s estimated error variance and modification index (Table S1 and Table S2 in Multimedia Appendix 2). Composite reliability values were greater than 0.6 and average variance extracted values were greater than 0.5, except those for the dimension *behavior* (Table 3).

The square roots of average variance extracted of the dimensions *attitude*, *subjective norms*, *intention*, and *perceived behavioral control* exceeded the related correlations (Table 4), indicating discriminant validity in the structures in this study [25]. The overall model achieved a good fit (GFI 0.991; CFI 0.992; adjusted GFI 0.987; RMSEA 0.029); therefore, the measurements and structural model were acceptable.

**Table 3:** Item reliability.

Dimension		Parameter significance estimation					Factor loading		Squared multiple correlation		Composite reliability		Average variance extracted
		Unstandardized estimate	SE	*t* value	*P* value	Standardized estimate							
**Attitude**										0.902		0.698	
	Q1	1.000				0.831		0.691					
	Q2	1.020	0.011	96.496	<.001	0.837		0.701					
	Q3	0.968	0.010	96.319	<.001	0.836		0.699					
	Q4	0.988	0.010	96.319	<.001	0.836		0.699					
**Subjective norms**										0.759		0.514	
	Q5	1.000				0.628		0.394					
	Q6	1.104	0.022	50.029	<.001	0.750		0.563					
	Q7	1.135	0.023	49.800	<.001	0.765		0.585					
**Perceived behavioral control**										0.910		0.671	
	Q8	1.000				0.814		0.663					
	Q9	0.976	0.012	84.038	<.001	0.761		0.579					
	Q10	1.008	0.010	99.196	<.001	0.860		0.740					
	Q11	1.017	0.011	94.843	<.001	0.832		0.692					
	Q12	0.996	0.011	93.580	<.001	0.824		0.679					
**Intention**										0.863		0.678	
	Q13	1.000				0.811		0.658					
	Q14	1.028	0.012	84.088	<.001	0.837		0.701					
	Q15	0.979	0.012	83.315	<.001	0.821		0.674					
**Behavior**										0.549		0.300	
	Q16	1.000				0.434		0.188					
	Q17	2.062	0.083	24.826	<.001	0.453		0.205					
	Q18	3.069	0.156	19.664	<.001	0.712		0.507					

**Table 4:** Construct validity.

Dimension	Average variance extracted	Dimension, correlation				
		Perceived behavioral control	Behavior	Intention	Subjective norms	Attitude
Perceived behavioral control	0.671	0.819	—^a^	—	—	—
Behavior	0.300	0.618	0.548	—	—	—
Intention	0.678	0.419	0.671	0.823	—	—
Subjective norms	0.514	0.406	0.601	0.683	0.717	—
Attitude	0.698	0.486	0.639	0.587	0.551	0.835

^a^Repeated correlation coefficients are omitted. The first occurrence value in each column of dimension is the square root of the average variance extracted for each latent variable.

### Hierarchical Moderator Regression Analysis

Annual household income, education, gender, health care occupation, chronic diseases, health self-assessment, and past vaccination behavior affected vaccination behavior (∆*R*^2^=0.121, *P*<.001); however, whether participants lived alone or not did not significantly affect vaccination behavior as a control variable (*P*=.08) (Table S3 in Multimedia Appendix 2). In block 3, the *attitude* * *perceived behavioral control* and *subjective norms* * *perceived behavioral control* terms significantly changed the model compared with block 2 (∆*R*^2^=0.003, *P*<.001).

In block 3, *perceived behavioral control* was positively and significantly correlated with vaccination behavior (β= 0.274, *P*<.001); however, *attitude* * *perceived behavioral control* (β=–0.052, *P*<.001) and *subjective norms* * *perceived behavioral control* (β=–0.028, *P*=.006) had reverse inhibitory effects on vaccination *behavior*. This not only indicates that *perceived behavioral control* moderates the impact of *attitude* on *behavior* and that of *subjective norms* on *behavior*, supporting hypotheses 5a and 5b, it also shows that there is a substitution relationship between *attitude*, *subjective norms*, and *perceived behavioral control* in their influence on vaccination *behavior*. Overall, *perceived behavioral control* weakens the positive effects of *attitude* and *subjective norms* on vaccination *behavior*, and when *perceived behavioral control* is low, the promotion effects of *attitude* and *subjective norms* on *behavior* are more pronounced, but with increases in *perceived behavioral control*, the positive effects of *attitude* and *subjective norm*s on *behavior* gradually decrease. Specifically, the slope describing effect of *attitude* on *behavior* will decrease by 0.050 SD and that of *subjective norms* on *behavior* will decrease by 0.021 SD when *perceived behavioral control* increases by 1 SD (Table 5; Table S3 in Multimedia Appendix 2).

**Table 5:** Model information in hierarchical moderator regression analysis.

Model	△*R*^2^	*F* value	*P* value
Block 1	0.121	151.760	<.001
Block 2	0.233	1192.784	<.001
Block 3	0.003	23.348	<.001

### Structural Equation Model of Vaccination Behavior

Both *attitude* (direct effect: β=0.493, *P*<.001) and *subjective norms* (direct effect: β=0.244, *P*<.001) showed significant positive associations with *behavior*. Hence, hypotheses 1 and 2 were confirmed. *Intention* also positively and significantly affected vaccination *behavior* (direct effect: β=0.462, *P*<.001); therefore, hypothesis 3 was confirmed. In addition, bootstrapping indicated that *intention* was present as a positive and significant mediator between *attitude* and vaccination *behavior* (indirect effect: β=0.159, *P*<.001). Similarly, the mediating effect between *subjective norms* and vaccination *behavior* was positive and significant (indirect effect β=0.258, *P*<.001); therefore, hypotheses 4a and 4b were confirmed (Table 6). Furthermore, the difference between the indirect effect of *attitude* on *behavior* and that of *subjective norms* on *behavior* was statistically significant (difference=–0.091, *P*<.001), which indicated that *subjective norms* had a greater influence on vaccination *behavior* than *attitude*.

**Table 6:** Direct, indirect, and total effects.

Effects		Unstandardized point estimate	SE	Bootstrapping			Bootstrapping, bias-corrected			*P* value
				95% CI lower	95% CI upper	95% CI lower		95% CI upper		
**Direct**										
	Attitude---Intention	0.344	0.021	0.303	0.384	0.303		0.384	<.001	
	Subjective norms---Intention	0.558	0.025	0.510	0.609	0.510		0.609	<.001	
	Intention---Behavior	0.462	0.030	0.404	0.522	0.403		0.521	<.001	
	Attitude---Behavior	0.493	0.029	0.437	0.550	0.437		0.551	<.001	
	Subjective norms---Behavior	0.244	0.032	0.182	0.307	0.182		0.307	<.001	
**Indirect**										
	Attitude---Behavior	0.159	0.014	0.132	0.187	0.132		0.188	<.001	
	Subjective norms---Behavior	0.258	0.019	0.222	0.298	0.222		0.299	<.001	
**Total**										
	Attitude---Behavior	0.652	0.030	0.594	0.710	0.594		0.710	<.001	
	Subjective norms---Behavior	0.501	0.030	0.443	0.562	0.443		0.562	<.001	

## Discussion

### Principal Results

Our findings support the hypothesis that intentions mediate vaccination behavior, attitudes, and subjective norms and addresses concern about the mediating process of intention in the theory of planned behavior [31], while also justifying initial theoretical claims that distal attitudes and subjective norms can influence behavior, through proximal intention mediators [32]. In particular, *perceived behavioral control* was found to be a moderator that influences the mediating processes *attitude*→*intention*→*behavior* and *subjective norms*→*intention*→*behavior*.

### Comparison With Prior Work

A large meta-analysis [15] of applications of theory of planned behavior showed that subjective norms were weak predictors of intention and behavior. Recent studies [33,34] that have used the theory of planned behavior empirically for health behavior have suggested that subjective norms are not the most critical predictor. In contrast, some argued that subjective norms are strong predictors [35,36]. Our findings are similar to those in [35,36], and the indirect effects in our study further show that subjective norms have greater impacts on behavior than attitudes. This indicates that decisions about health issues are more likely to be influenced by social surroundings. Debate about whether subjective norms have a strong or weak influence on behavior might due to differences in the types of behaviors that have been targeted. In contrast to high-frequency health behaviors, such as exercise, smoking cessation, when making decisions about vaccination, people expect to be counseled by someone close to them (eg, family members and close friends [37]), or someone they trust (eg, a physician [38]). Additionally, socially desirable responses likely contributed to our finding that attitudes have less explanatory power for behavior. Socially desirable responses are defined as the tendency to give a positive self-description, which is relatively common for potentially sensitive problems. Korn [39] suggested that vaccination is a prosocial behavior and demonstrated that it is part of the social contract by showing that there is significant intergroup bias in vaccinated and unvaccinated cohorts. Therefore, it is reasonable that participants glorified attitudes toward the vaccine and responded positively to the investigation, rather than providing their true thoughts.

Our findings also show that high *perceived behavioral control* weakens the effect between *attitude*, *subjective norms*, and vaccination *behavior*, using *intention* as a mediator. Additionally, *perceived behavioral control* has an alternative relationship with *attitude* and *subjective norms*, when present as a moderator in the model. These findings extend those in existing literature on perceived behavioral control. However, perceived behavioral control was considered to be a positive predictor of intentions in many existing studies [40-42]. Our findings (block 2 in the hierarchical regression analysis) also confirm this view. It is important to consider that if a given behavior is seen as positive and implementable, people tend to engage in that behavior. However, the gap between intentions and behavior exists precisely because intention is not sufficient to ensure that a person converts ideas into actual behavior, due to the limitations of actual capabilities. Thus, we find that the mechanisms by which *perceived behavioral control* affect *intention* and *behavio*r are different. In other words, the influence of *perceived behavioral control* on *intention* is as positive as *attitude* and *subjective norms*. However, when using *perceived behavioral control* to predict behavior, it should be thought of as actual behavior control [23]. Therefore, it is more appropriate to use *perceived behavioral control* as a moderating variable to influence the mediating effect of *intention* on *behavior*.

### Practical Applications

The moderated mediation model provides evidence for the practical application of theory of planned behavior in vaccination behavior. Future interventions for people who are unwilling to be vaccinated or subjectively postpone vaccination should not only focus on changing their negative attitudes toward the vaccine but should also pay attention to the ideas of their families and close friends about vaccination and intervention. This will facilitate positive intentions to get vaccinated. In a study [43] that explored factory workers' vaccination behavior using the theory of planned behavior, it was also shown that a positive attitude and the support of health care workers, relatives, and friends can contribute to individuals getting vaccinated, and in another study [44], it was found that trusted individuals had a unique influence on young women in encouraging them to get their HPV vaccination. In addition, health authorities may need to take measures such as communicating risk or giving rewards for vaccinations to improve influenza and HPV vaccine uptake.

### Limitations

This study was conducted in the form of a web-based survey; thus, convenience sampling might have caused selection bias. Additionally, we used a cross-sectional survey; thus, information on respondents' vaccine-related behavior at the time of the survey was used in behavior dimension. For this reason, the values of composite reliability and average variance extracted for the *behavior* dimension were below the ideal value. However, the overall model fit was ideal, with GFI, adjusted GFI, and CFI >0.9 and RMSEA <0.06.

Data from 1 month after the survey showed that the actual COVID-19 vaccination rate (having completed at least one shot), in China was 74% [45], approximating the self-reported behavioral data in this study (8614/9924, 86.8%). Although the self-reported data of this study are credible, cautious interpretation of the sample’s representativeness of vaccination behavior is needed.

The *behavior* dimension was measured with 3 items—self-reported COVID-19 vaccination behavior and influenza vaccination behavior, as well as overall vaccine hesitancy. This is because according to the schedule of immunization in China [46], there are 5 main vaccines for adults 18 to 59 years of age—COVID-19, influenza, HPV, hepatitis B, and rabies. Yearly influenza and the recent COVID-19 vaccine were the used to examine vaccination behavior because the administration of the other 3 types are limited—hepatitis B vaccinations are valid for a long period, rabies vaccinations are for emergency use after possible exposure, and HPV vaccinations are mainly target the female population in China.

### Conclusions

Our findings showed that subjective norms have a stronger influence than attitudes on this particular vaccination practice. Moreover, perceived behavioral control not only is a positive facilitator of intention but also has an alternative relationship with attitudes and subjective norms. As a moderator, perceived behavioral control conversely weakens the positive effects of attitudes and subjective norms on vaccination behaviors. When perceived behavioral control is low, the positive influence of attitudes and subjective norms are evident. However, when perceived behavioral control is high, the positive effects of attitudes and subjective norms gradually decrease. In particular, as a moderator, perceived behavioral control has more predictive power for vaccination behavior.

## Appendix

Multimedia Appendix 1 Questionnaire.

Multimedia Appendix 2 Supplementary material.

## Abbreviations

CFI: comparative fit index
GFI: goodness-of-fit index
HPV: human papillomavirus
RMSEA: root mean square error of approximation
