# Supplementary material for: Vaccination Intention and Behavior of the General Public in China: Cross-sectional Survey and Moderated Mediation Model Analysis
Source: JMIR Public Health Surveill. 2022 Jun 20;8(6):e34666. doi: 10.2196/34666 (PMC9253970; doi:10.2196/34666)
Supplement: Multimedia Appendix 1 [file publichealth_v8i6e34666_app1.pdf]

Multimedia Appendix 1

Supplementary Figure S1. The Screenshots of the Original Questionnaire
Supplementary Materia l. Chinese and English versions of the questionnaire "Survey of Chinese Residents' Attitudes to Vaccination"

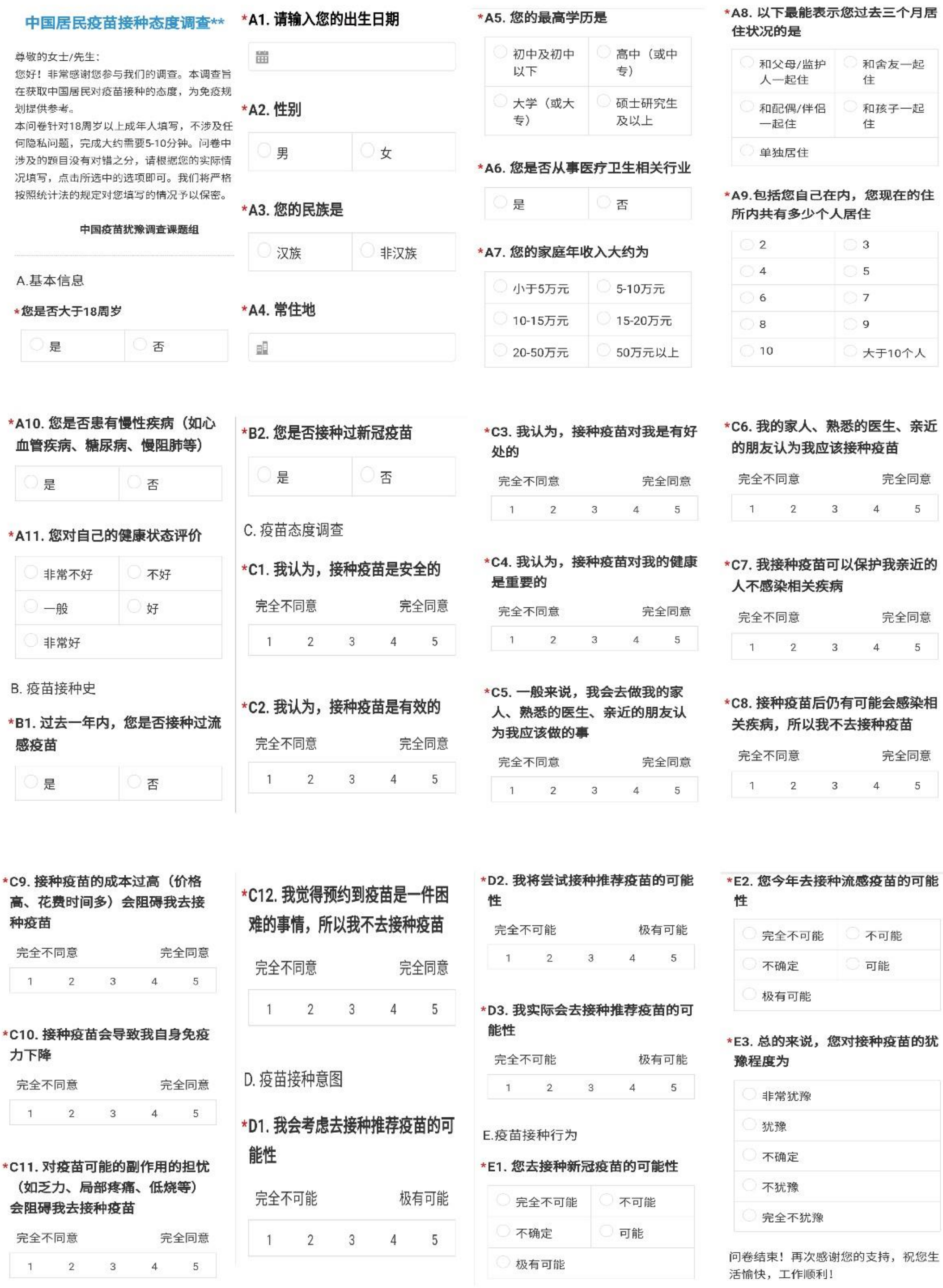

中国居民疫苗接种态度调查**

尊敬的女士/先生：
您好！非常感谢您参与我们的调查。本调查旨在获取中国居民对疫苗接种的态度，为免疫规划提供参考。
本问卷针对18周岁以上成年人填写，不涉及任何隐私问题，完成大约需要5-10分钟。问卷中涉及的题目没有对错之分，请根据您的实际情况填写，点击所选中的选项即可。我们将严格按照统计法的规定对您填写的情况予以保密。

中国疫苗犹豫调查课题组

A.基本信息

*您是否大于18周岁
是 否

*A1. 请输入您的出生日期

*A2. 性别
男 女

*A3. 您的民族是
汉族 非汉族

*A4. 常住地

*A5. 您的最高学历是
初中及初中以下 高中（或中专）
大学（或大专） 硕士研究生及以上

*A6. 您是否从事医疗卫生相关行业
是 否

*A7. 您的家庭年收入大约为
小于5万元 5-10万元
10-15万元 15-20万元
20-50万元 50万元以上

*A8. 以下最能表示您过去三个月居住状况的是
和父母/监护人一起住 和舍友一起住
和配偶/伴侣一起住 和孩子一起住
单独居住

*A9.包括您自己在内，您现在的住所内共有多少个人居住
2 3
4 5
6 7
8 9
10 大于10个人

*A10. 您是否患有慢性疾病（如心血管疾病、糖尿病、慢阻肺等）
是 否

*A11. 您对自己的健康状态评价
非常不好 不好
一般 好
非常好

B. 疫苗接种史

*B1. 过去一年内，您是否接种过流感疫苗
是 否

*B2. 您是否接种过新冠疫苗
是 否

C. 疫苗态度调查

*C1. 我认为，接种疫苗是安全的
完全不同意 完全同意
1 2 3 4 5

*C2. 我认为，接种疫苗是有效的
完全不同意 完全同意
1 2 3 4 5

*C3. 我认为，接种疫苗对我是有好处的
完全不同意 完全同意
1 2 3 4 5

*C4. 我认为，接种疫苗对我的健康是重要的
完全不同意 完全同意
1 2 3 4 5

*C5. 一般来说，我会去做我的家人、熟悉的医生、亲近的朋友认为我应该做的事
完全不同意 完全同意
1 2 3 4 5

*C6. 我的家人、熟悉的医生、亲近的朋友认为我应该接种疫苗
完全不同意 完全同意
1 2 3 4 5

*C7. 我接种疫苗可以保护我亲近的人不感染相关疾病
完全不同意 完全同意
1 2 3 4 5

*C8. 接种疫苗后仍有可能会感染相关疾病，所以我不去接种疫苗
完全不同意 完全同意
1 2 3 4 5

*C9. 接种疫苗的成本过高（价格高、花费时间多）会阻碍我去接种疫苗
完全不同意 完全同意
1 2 3 4 5

*C10. 接种疫苗会导致我自身免疫力下降
完全不同意 完全同意
1 2 3 4 5

*C11. 对疫苗可能的副作用的担忧（如乏力、局部疼痛、低烧等）会阻碍我去接种疫苗
完全不同意 完全同意
1 2 3 4 5

*C12. 我觉得预约到疫苗是一件困难的事情，所以我不去接种疫苗
完全不同意 完全同意
1 2 3 4 5

D. 疫苗接种意图

*D1. 我会考虑去接种推荐疫苗的可能性
完全不可能 极有可能
1 2 3 4 5

*D2. 我将尝试接种推荐疫苗的可能性
完全不可能 极有可能
1 2 3 4 5

*D3. 我实际会去接种推荐疫苗的可能性
完全不可能 极有可能
1 2 3 4 5

E.疫苗接种行为

*E1. 您去接种新冠疫苗的可能性
完全不可能 不可能
不确定 可能
极有可能

*E2. 您今年去接种流感疫苗的可能性
完全不可能 不可能
不确定 可能
极有可能

*E3. 总的来说，您对接种疫苗的犹豫程度为
非常犹豫
犹豫
不确定
不犹豫
完全不犹豫

问卷结束！再次感谢您的支持，祝您生活愉快，工作顺利！

Supplementary Figure S1. The Screenshots of the Original Questionnaire

Survey of Chinese Residents' Attitudes to Vaccination

Dear Ms./Mr.

Hello! Thank you very much for taking part in our survey. The purpose of this survey is to obtain Chinese residents' attitudes towards vaccination and to provide reference for immunization planning.
This questionnaire is for adults over the age of 18 and does not involve any privacy issues. It takes about 5-10 minutes to complete. There is no right or wrong answer to the questions involved in the questionnaire, so please fill in according to your actual situation and click the selected option. We will keep the information you fill in confidential in strict accordance with the provisions of the Statistics Law.

China Vaccine Hesitant Investigation Team

Part A. Demographic Information

Are you over 18 years old?

□Yes □No (skip to the end of the questionnaire)

A1. Date of Birth: ______

A2. Gender:

□ Male □ Female

A3. ethnic group:

□ Han ethnic group □ Non-Han ethnic group

A4. Permanent residence: _________ (city), _________ (province)

A5. Education level:

□ Junior high school or below □ High school

□ College or equivalent □Master's diploma or above

A6. Are you engaged in medical and health related industries: □Yes □No

A7. Annual household income:

□ Less than $8,000 □$8,000-16,000 □$16,000-24,000

□ $24,000-32,000 □$32,000-80,000 □$80,000 or more

A8. What is the best indicator of your living condition?

□ Living with parent/guardian □ Living with roommate □ Living with spouse/partner

□ Living with children □ Living alone (skip to A10)

A9. Including yourself, how many people live in your current residence?

□2 □3 □4 □5 □6 □7 □8 □9 □10 □More than 10 people

A10. Do you suffer from chronic diseases (such as cardiovascular disease,

diabetes, chronic obstructive pulmonary disease, etc.)?

□Yes □No

A11. Your evaluation of your own health status:

□Very bad □Bad □General □Well □Very well

Part B. Vaccination History

B1. In the past year, have you ever been vaccinated against influenza?

□Yes □No

B2. Have you ever been vaccinated against COVID-19?

□Yes □No

B3. Have you received HPV vaccine (human papillomavirus vaccine) [depending on A2]?

□Yes □No

Part C. Vaccination Attitude Survey

C1. I think that vaccination is safe.

strongly disagree：__1__: __2__: __3__: __4__: __5__: strongly agree

C2. I think that vaccination is effective.

strongly disagree：__1__: __2__: __3__: __4__: __5__: strongly agree

C3. I think that vaccination is beneficial.

strongly disagree：__1__: __2__: __3__: __4__: __5__: strongly agree

C4. I think that vaccination is important.

strongly disagree：__1__: __2__: __3__: __4__: __5__: strongly agree

C5. Did my family / doctors / close friends think I should be vaccinated?

strongly disagree：__1__: __2__: __3__: __4__: __5__: strongly agree

C6. Will I do what they think I should do?

strongly disagree：__1__: __2__: __3__: __4__: __5__: strongly agree

C7. Can vaccination can protect close relatives from relevant vaccine protected diseases?

strongly disagree：__1__: __2__: __3__: __4__: __5__: strongly agree

C8. The possibility of still being infected after vaccination would discourage me from getting vaccinated.

strongly disagree：__1__: __2__: __3__: __4__: __5__: strongly agree

C9. The exorbitant cost of vaccinating would stop me from getting vaccinated.

strongly disagree：__1__: __2__: __3__: __4__: __5__: strongly agree

C10. Vaccination causes a decline in autoimmunity.

strongly disagree： __1__: __2__: __3__: __4__: __5__: strongly agree

C11. Concerns about side effects of the vaccine stop me from getting vaccinated.

strongly disagree： __1__: __2__: __3__: __4__: __5__: strongly agree

C12. Difficulty in obtaining an appointment for vaccination would prevent me from getting vaccinated.

strongly disagree： __1__: __2__: __3__: __4__: __5__: strongly agree

Part D. Vaccination Intention

D1. The possibility of considering getting vaccinated.

completely impossible： __1__: __2__: __3__: __4__: __5__: completely possible

D2. The possibility of trying to get vaccinated.

completely impossible： __1__: __2__: __3__: __4__: __5__: completely possible

D3. The possibility of actually getting vaccinated.

completely impossible： __1__: __2__: __3__: __4__: __5__: completely possible

Part E. Vaccination Behavior

E1. How likely are you to go for a COVID-19 vaccine?

completely impossible： __1__: __2__: __3__: __4__: __5__: completely possible

E2. What is the possibility of getting an influenza shot this year?

completely impossible： __1__: __2__: __3__: __4__: __5__: completely possible

E3. What is the level of hesitation about vaccinating?

very hesitant： __1__: __2__: __3__: __4__: __5__: no hesitation at all

中国居民疫苗接种态度调查

尊敬的女士/先生：

您好！非常感谢您参与我们的调查。本调查旨在获取中国居民对疫苗接种的态度，为免疫规划提供参考。

本问卷针对 18 周岁以上成年人填写，不涉及任何隐私问题，完成大约需要 5-10 分钟。问卷中涉及的题目没有对错之分，请根据您的实际情况填写，点击所选中的选项即可。我们将严格按照统计法的规定对您填写的情况予以保密。

中国疫苗犹豫调查课题组

A. 基本信息

您是否大于 18 周岁

□是 □否（跳至问卷结束）

A1. 您的出生日期：________年________月

A2. 性别：□男 □女

A3. 您的民族是：□汉族 □非汉族

A4. 常住地：________省________市

A5. 您的最高学历是：□初中及初中以下 □高中 （或中专）

□大学（或大专） □硕士研究生及以上

A6. 您是否从事医疗卫生相关行业： □是 □否

A7. 您的家庭年收入大约为：□小于 5 万元 □5-10 万元 □10-15 万元

□15-20 万元 □20-50 万元 □50 万元以上

A8. 以下最能表示您过去三个月居住状况的是：

□和父母/监护人一起住 □和舍友一起住 □和配偶/伴侣一起住

□和孩子一起住 □单独居住（跳至 A10）

A9. 包括您自己在内，您现在的住所内共有多少人居住？

□2 □3 □4 □5 □6 □7 □8 □9 □10 □大于 10 个人

A10. 您是否患有慢性疾病（如心血管疾病、糖尿病、慢阻肺等）：□是 □否

A11. 您对自己的健康状态评价：□非常不好 □不好 □一般 □好 □非常好

B. 疫苗接种史

B1. 过去一年内，您是否接种过流感疫苗：□是 □否

B2. 您是否接种过新冠疫苗：□是 □否

B3. 您是否接种过 HPV 疫苗（宫颈癌疫苗）[依赖于 A2 选项]：□是 □否

C. 疫苗态度调查

C1. 我认为，接种疫苗是安全的：

完全不同意：__1__:__2__:__3__:__4__:__5__:完全同意

C2. 我认为，接种疫苗是有效的：

完全不同意：__1__:__2__:__3__:__4__:__5__:完全同意

C3. 我认为，接种疫苗对我是有好处的：

完全不同意：__1__:__2__:__3__:__4__:__5__:完全同意

C4. 我认为，接种疫苗对我的健康是重要的：

完全不同意：__1__:__2__:__3__:__4__:__5__:完全同意

C5. 我的家人、熟悉的医生、亲近的朋友认为我应该接种疫苗：

完全不同意：__1__:__2__:__3__:__4__:__5__:完全同意

C6. 一般来说，我会去做我的家人、熟悉的医生、亲近的朋友认为我应该做的事：

完全不同意：__1__:__2__:__3__:__4__:__5__:完全同意

C7. 我接种疫苗可以保护我亲近的人不感染相关疾病:

完全不同意：__1__:__2__:__3__:__4__:__5__:完全同意

C8. 接种疫苗后仍有可能会感染相关疾病，所以我不去接种疫苗：

完全不同意：__1__:__2__:__3__:__4__:__5__:完全同意

C9. 接种疫苗的成本过高（价格高、花费时间多）会阻碍我去接种疫苗：

完全不同意：__1__:__2__:__3__:__4__:__5__:完全同意

C10. 接种疫苗会导致我自身免疫力下降：

完全不同意：__1__:__2__:__3__:__4__:__5__:完全同意

C11：对疫苗可能的副作用的担忧（如乏力、局部疼痛、低烧等）会阻碍我去接种疫苗：

完全不同意：__1__:__2__:__3__:__4__:__5__:完全同意

C12. 我觉得预约到疫苗是一件困难的事情，所以我不去接种疫苗：

完全不同意：__1__:__2__:__3__:__4__:__5__:完全同意

D. 疫苗接种意图

D1: 我会考虑去接种推荐疫苗的可能性有多大：

完全不可能：__1__:__2__:__3__:__4__:__5__:极有可能

D2: 我将尝试接种推荐疫苗的可能性有多大：

完全不可能：__1__:__2__:__3__:__4__:__5__:极有可能

D3: 我实际会去接种推荐疫苗的可能性有多大：

完全不可能：__1__:__2__:__3__:__4__:__5__:极有可能

E.疫苗行为

E1. 您去接种新冠疫苗的可能性(已接种人群跳过此题):

完全不可能：__1__:__2__:__3__:__4__:__5__:极有可能

E2. 您今年去接种流感疫苗的可能性：

完全不可能：__1__:__2__:__3__:__4__:__5__:极有可能

E3. 总的来说，您对接种疫苗的犹豫程度为：

□非常犹豫 □犹豫 □不确定 □不犹豫 □完全不犹豫
