# Supplementary material for: Vaccination Intention and Behavior of the General Public in China: Cross-sectional Survey and Moderated Mediation Model Analysis
Source: JMIR Public Health Surveill. 2022 Jun 20;8(6):e34666. doi: 10.2196/34666 (PMC9253970; doi:10.2196/34666)
Supplement: Multimedia Appendix 2 [file publichealth_v8i6e34666_app2.pdf]

Multimedia Appendix 2

Supplementary Table S1. The Estimate of Variances
Supplementary Table S2. The Modification Indices of Items
Supplementary Table S3. Variable Coefficients of Block 3 in Hierarchical Moderator Regression Analysis
Supplementary Material 2. The policy on COVID-19 vaccination, HPV vaccination and influenza vaccination in China

Supplementary Table S1. The Estimate of Variances

Dimension	Item	Variances	
		Estimate	p-value
Attitude	Q1	0.135	<0.000
	Q2	0.133	<0.000
	Q3	0.121	<0.000
	Q4	0.126	<0.000
Subjective norms	Q5	0.515	<0.000
	Q6	0.318	<0.000
	Q7	0.306	<0.000
Perceived behavior control	Q8	0.605	<0.000
	Q9	0.821	<0.000
	Q10	0.423	<0.000
	Q11	0.545	<0.000
	Q12	0.555	<0.000
Intention	Q13	0.206	<0.000
	Q14	0.179	<0.000
	Q15	0.183	<0.000
Behavior	Q16	0.281	<0.000
	Q17	1.076	<0.000
	Q18	0.598	<0.000

Supplementary Table S2. The Modification Indices of Items

Pathway	M.I.	Par Change
e1<-->e3	52.650	0.012
e2<-->e3	68.739	-0.013
e4<-->e1	71.528	-0.014
e4<-->e2	50.681	0.012
e10<-->e9	83.430	-0.065
e10<-->e8	55.228	0.046
e11<-->e9	62.042	0.062
e11<-->e8	83.430	-0.049

Supplementary Table S3. Variable Coefficients of Block 3 in Hierarchical Moderator Regression Analysis

Variables	Unstd. [a]		Std. [c]	t-value	p-value
	β	S.E. [b]			
Constant	-1.242	0.097	-	-12.820	<0.001
Gender	-0.060	0.018	-0.030	-3.315	0.001
Healthcare occupation	0.076	0.019	0.035	3.898	<0.001
Chronic diseases	0.187	0.028	0.063	6.771	<0.001
Health self-assessment	0.152	0.009	0.146	17.143	<0.001
Influenza vaccination history	0.092	0.005	0.167	18.260	<0.001
HPV vaccination history	0.030	0.008	0.036	4.021	<0.001
Annual household income	0.025	0.010	0.022	2.546	0.011
Education	0.057	0.016	0.031	3.538	<0.001
Main living condition	-0.053	0.030	-0.014	-1.743	0.081
ATT [d]	0.188	0.011	0.188	17.781	<0.001
SN [e]	0.188	0.009	0.188	20.013	<0.001
PBC [f]	0.274	0.010	0.274	28.550	<0.001
ATT*PBC	-0.052	0.010	-0.048	-5.109	<0.001
SN*PBC	-0.028	0.010	-0.024	-2.761	0.006

a. Unstd. is the abbreviation of unstandardized estimate

b. S.E. is the abbreviation of standard error

c. Std. is the abbreviation of standardized estimate

d. ATT is the abbreviation of attitude

e. SN is the abbreviation of subject norms

f. PBC is the abbreviation of perceived behavior control

Supplementary material 2. The policy on COVID-19 vaccination, HPV vaccination and influenza vaccination in China

COVID-19 vaccination [1,2,3]

There are 5 manufacturers of COVID-19 vaccines approved for conditional marketing or emergency use in China. Among them, three inactivated vaccines and one adenovirus vector vaccine have been conditionally approved for marketing; in addition, one recombinant neo-coronavirus vaccine (CHO cells) has been approved for emergency use. The vaccines are free of charge for people with no contraindications, with informed consent and on a voluntary basis.

People aged 18 years and above

All of the above vaccines are available to people over 18 years of age. Currently, two doses of inactivated COVID-19 vaccines are required; the interval between the first and second doses should be 3 weeks or more, and the second dose should be completed as early as possible within 8 weeks after the first dose. Adenovirus vector vaccine requires 1 dose. Recombinant subunit vaccine requires 3 doses; the interval between the first and second doses and between the second and third doses is recommended to be 4 weeks and above. The second dose should be completed within 8 weeks of the first dose, and the third dose within 6 months of the first dose, if possible.

People aged 3-17 years

The only technical route of vaccine for emergency use in the 3-17 years old population is inactivated vaccine, which requires 2 doses at an interval of 3-8 weeks.

Booster immunization

For booster immunizations, three inactivated vaccines and one adenovirus vector vaccine are approved for implementation. The booster immunization will be implemented in people aged 18 years and above who have received the above vaccines for the full 6 months, according to the needs of epidemic prevention and control. The booster immunization with the new crown vaccine is still implemented in accordance with the free vaccination policy for residents.

[1] China CDC. Technical Guide to Vaccination against New Coronavirus (First Edition). URL:http://www.nhc.gov.cn/jkj/s3582/202103/c2febfd04fc5498f916b1be080905771.shtml [accessed 2022-02-23]
[2] National Health Commission. Questions and Answers about COVID-19 Vaccination. URL:http://www.nhc.gov.cn/xcs/yqfkdt/202111/67a59e40580d4b4687b3ed738333f6a9.shtml[accessed 2022-02-23]
[3] National Health Commission. Questions and answers about theCOVID-19 vaccination for people aged 3-11 years
URL:http://www.nhc.gov.cn/xcs/yqfkdt/202111/79103c66c2de404b8e50583816f5e31e.shtml[accessed 2022-02-23]

Influenza vaccination [1]

The influenza vaccines approved for marketing in China are trivalent inactivated influenza vaccine (IIV3), quadrivalent inactivated influenza vaccine (IIV4) and trivalent live attenuated influenza vaccine (LAIV3), IIV3 includes both cracked and subunit vaccines, IIV4 is a cracked vaccine and LAIV is an attenuated vaccine. Influenza vaccine is a non-immunization vaccine in most areas of China, and is voluntary and self-funded.

[1] Working Group on Influenza Vaccine, National Technical Working Group on Immunization Planning. Technical guidelines for influenza vaccination in China (2021-2022)[J]. Chinese Journal of Epidemiology,2021,42(10):1722-1749.

HPV vaccination [1]

Four HPV vaccine products have been marketed in China, namely Glaxo's bivalent HPV vaccine (Cirex), Merck Sharp & Dohme's quadrivalent vaccine (Gadaxiu-4) and nine-valent vaccine (Gadaxiu-9), and Xiamen Wantai's domestic bivalent HPV vaccine (Xin Ke Ning), and the four vaccines were approved for marketing or conditionally approved for marketing by China's drug regulatory authorities in July 2016, May 2017, April 2018, and December 2019, respectively.

Promote the inclusion of HPV vaccines in the priority review and approval process by the State Drug Administration and other authorities to improve the accessibility of HPV vaccines. And on the basis of scientific evidence, promote pilot first, guide localities to explore effective models and multiple funding channels for HPV vaccination, and encourage areas with mature conditions to include HPV vaccine in local immunization planning.

[1] National Health Commission. Letter of Response to Proposal No. 4216 (Medical and Sports No. 467) of the Fourth Session of the 13th National Committee of the Chinese People's Political Consultative Conference URL:http://www.nhc.gov.cn/wjw/tia/202112/af51b14c0ca04799b55b8322559751ac.shtml [accessed 2022-02-23]
